# Development of Non-Immunosuppressive FK506 Derivatives as Antifungal and Neurotrophic Agents

**DOI:** 10.4014/jmb.1911.11008

**Published:** 2019-11-18

**Authors:** Jin A Jung, Yeo Joon Yoon

**Affiliations:** Department of Chemistry and Nanoscience, Ewha Womans University, Seoul 03760, Republic of Korea

**Keywords:** FK506, biosynthesis, antifungal activity, neurotrophic activity

## Abstract

FK506, also known as tacrolimus, is a clinically important immunosuppressant drug and has promising therapeutic potentials owing to its antifungal, neuroprotective, and neuroregenerative activities. To generate various FK506 derivatives, the structure of FK506 has been modified by chemical methods or biosynthetic pathway engineering. Herein, we describe the mode of the antifungal action of FK506 and the structure–activity relationship of FK506 derivatives in the context of immunosuppressive and antifungal activities. In addition, we discuss the neurotrophic mechanism of FK506 known to date, along with the neurotrophic FK506 derivatives with significantly reduced immunosuppressive activity. This review suggests the possibility to generate novel FK506 derivatives as antifungal as well as neuroregenerative/neuroprotective agents.

## Introduction

FK506, an FDA-approved immunosuppressant, is a 23membered macrocyclic polyketide and has been used to prevent organ transplant rejection. In 1984, FK506 was first isolated from *Streptomyces tsukubanesis* No. 9993 found in the soil at Tsukuba, Japan [1-3]. Since then, several *Streptomyces* species have been discovered as FK506 producers [4-6].

Previous studies reported that FK506 exhibited various therapeutic activities, such as antifungal [7, 8], neuroprotective [9], neuroregenerative [10], and immunosuppressive activities [1, 2]. The activity of FK506 immunosuppression results from inhibition of T-cell proliferation. FK506 interacts with an FK506-binding protein (FKBP12) to form the FKBP12–FK506 complex, which inhibits calcineurin (CaN). The resulting FKBP12–FK506–CaN complex suppresses interleukin-2 (IL-2) production and prevents T-cell activation [11, 12]. The mechanism of antifungal action of FK506 is very similar to its immunosuppression mechanism. The CaN pathway plays essential roles in the growth and pathogenesis of fungal pathogens, and thus, the inhibition of fungal CaN can lead to the antifungal activity [8]. In contrast, neuronal property of FK506 may not be directly related to CaN; however, FKBPs associated with steroid hormone receptors are certainly involved in the neurotrophic mechanism of FK506 [10]. To develop FK506 as a novel antifungal or neurotrophic agent, a significant reduction in its immunosuppressive activity is critical.

**Fig. 1:**
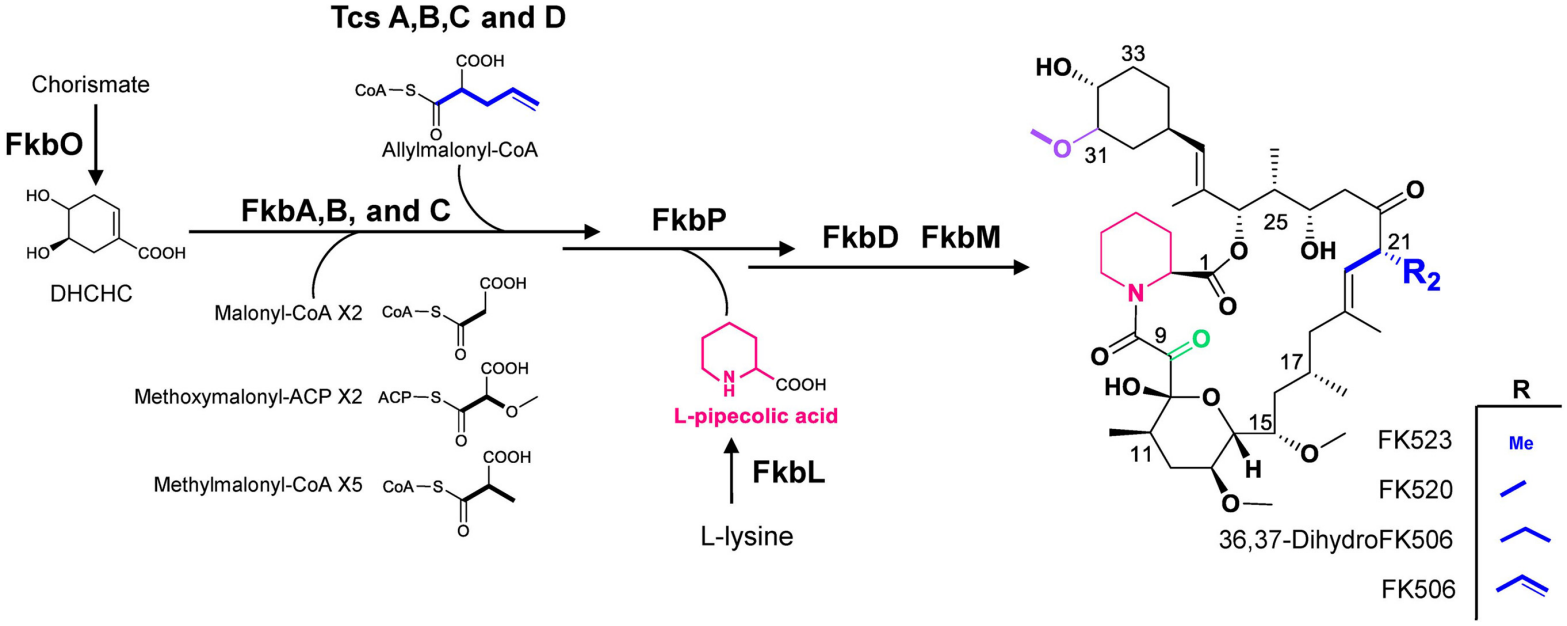
The biosynthesis of FK506 and its derivatives with modified C21 side chains.

FK506 is biosynthesized through a hybrid system of a type I modular polyketide synthetase (PKS) and non-ribosomal peptide synthetase (NRPS) (Fig. 1). Biosynthesis of FK506 core is initiated by the formation of (*4R,5R*)-4,5dihydroxycyclohex-1-enecarboxylic acid (DHCHC), which is derived from the chorismate by chorismatase, which is encoded by *fkbO* gene in the FK506 biosynthetic gene cluster [13]. The starter unit is extended by ten condensation steps with two malonyl-CoA, two methoxymalonyl-ACP (acyl carrier protein), five methylmalonyl-CoA, and an allymalonyl-CoA/ACP. The products of *tcsA*, *tcsB*, *tcsC*, and *tcsD* in the FK506-producing strain are involved in the biosynthesis of the unique extender unit allylmalonyl-CoA. Trans-2-pentenyl-ACP is generated by TcsB (β-ketoacyl synthase) and TcsA, which consists of acyl transferase (AT) and ACP didomain, in conjunction with fatty acid synthase pathway. TcsC (crotonyl-CoA carboxylase/reductase) and TcsD (dehydrogenase) catalyze reductive carboxylation reaction and double bond formation, respectively. The allylmalonyl–CoA/ACP is loaded onto the AT domain of module 4 (AT4) [14]. Particularly, AT4 has broad specificity toward various extender units, resulting in the generation of FK506 derivatives with various C21 side chains. The linear chain obtained by the elongation of PKS is incorporated with L-pipecolate derived from L-lysine by FkbL (lysine cyclodeaminase) [15] and cyclized by NRPS FkbP to form the macrolactone ring. The ring of FK506 requires final post-PKS modification steps, including C9oxidation by FkbD and C31-O-methylation by FkbM [16]. Based on this biosynthetic pathway of FK506, various FK506 derivatives were generated by combinatorial biosynthesis or biosynthetic pathway engineering [4].

Herein, we briefly cover the mode of antifungal and neurotrophic action of FK506. In addition, examples of antifungal and neurotrophic FK506 derivatives with reduced immunosuppressive activity are presented.

## Antifungal Mechanism of FK506 against Human Fungal Pathogens

**Fig. 2:**
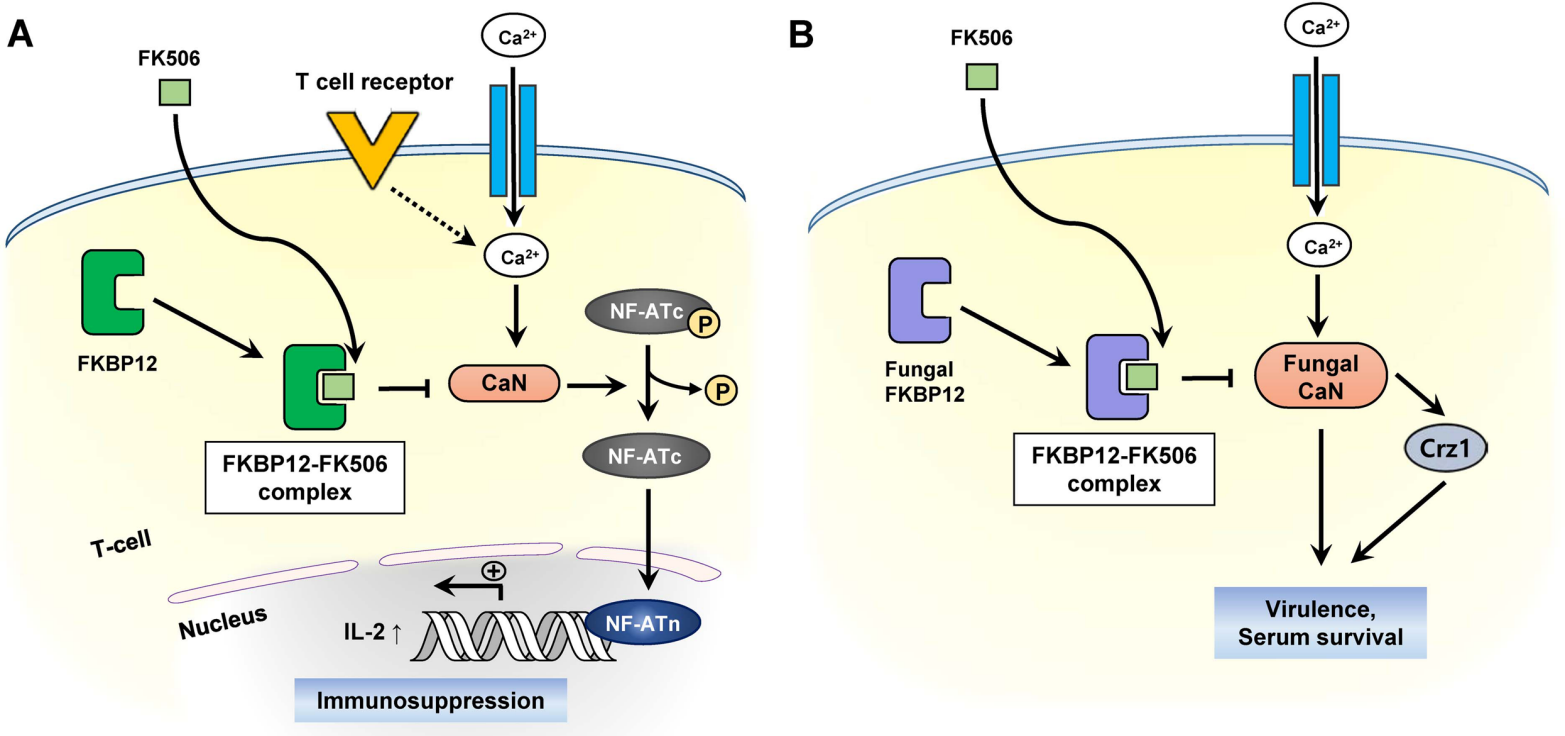
(**A**) Mechanism of the immunosuppressive action of FK506. (**B**) Antifungal action of FK506 against fungal pathogens. Crz1: Calcineurin-responsive zinc finger 1.

FK506 exerts antifungal activity and immunosuppressive activity by inhibiting CaN through the formation of FKBP12–FK506 binary complex [17] (Fig. 2). CaN, the Ca^2+^/ calmodulin-dependent protein phosphatase, is a heterodimer composed of catalytic A subunit and regulatory B subunit [18]. In addition to its role in T-cell proliferation in humans, it plays a pivotal role in viability, cell wall synthesis, and ion homeostasis in yeast [17-19]. CaN pathway is also essential for the survival of human fungal pathogens, including *Cryptococcus neoformans*, *Candida albicans*, and *Aspergillus fumigatus* [8]. *C. neoformans* is the most common cause of major opportunistic infections. The incidence of this infection can lead to cryptococcal meningitis, a common fatal disease [20, 21]. *C. albicans* is an invasive fungal pathogen, which causes superficial mucosal infection or candidemia due to a bloodstream infection with *Candida* [22, 23]. In case of *A. fumigatus*, severe infections, including invasive aspergillosis and severe asthma, occur in immunocompromised patients [24]. It was found that inactivation of CaN inhibited the growth of human fungal pathogens and abolished the antifungal activity of FK506 [23, 25, 26].

FK506 has potent antifungal activity against *C. neoformans* at 37°C. CaN encoded by CNA1 and CNB1 is essential for the virulence of *C. neoformans* [20, 25]. Disruption of genes encoding the catalytic A subunit or regulatory B subunit of CaN in *C. neoformans* (Cna1 or Cnb1) abolished the growth of *C. neoformans* at 37°C but maintained growth at 24°C [20, 25]. Unlike *C. neoformans*, the inhibition of CaN by FK506 in C.albicans is not temperature-sensitive. The *C. albicans* CaNmutant strains Δ*cmp1* (Δ*cna1*) or Δ*cnb1*, which lack catalyticA or regulatory B subunit, respectively, were not defunct atany temperature [23, 27]. However, CaN is required forsurvival in the presence of serum [23, 28]. The combinationof FK506 with fluconazole showed synergistic antifungalactivity by inhibiting the virulence of *C. albicans* in serumthrough the inactivation of CaN [29]. CaN is of particularimportance for filament growth in *C. neoformans* but not in *C. albicans*. In case of *A. fumigatus*, FK506 exhibits antifungalactivity against *A. fumigatus* regardless of growthtemperature, either alone or with caspofungin [30, 31]. In *A. fumigatus*, CaN plays an important role in hyphal growth [24]. Δ*calA* (Δ*cnaA*), *A. fumigatus* CaN catalytic A subunit mutant, was not affected by various temperatures, and led to decrease filamentation following defective hyphal growth [8, 24, 26]. Taken together, CaN plays a important role in fungal pathogens, and FK506 exhibits antifungal activity by inhibiting fungal CaNs. Although FK506 has potent antifungal activity against human fungal pathogens, it is necessary to remove its immunosuppressive property to develop FK506 as a new antifungal agent.

## Antifungal Activity of FK506 Analogs

**Fig. 3:**
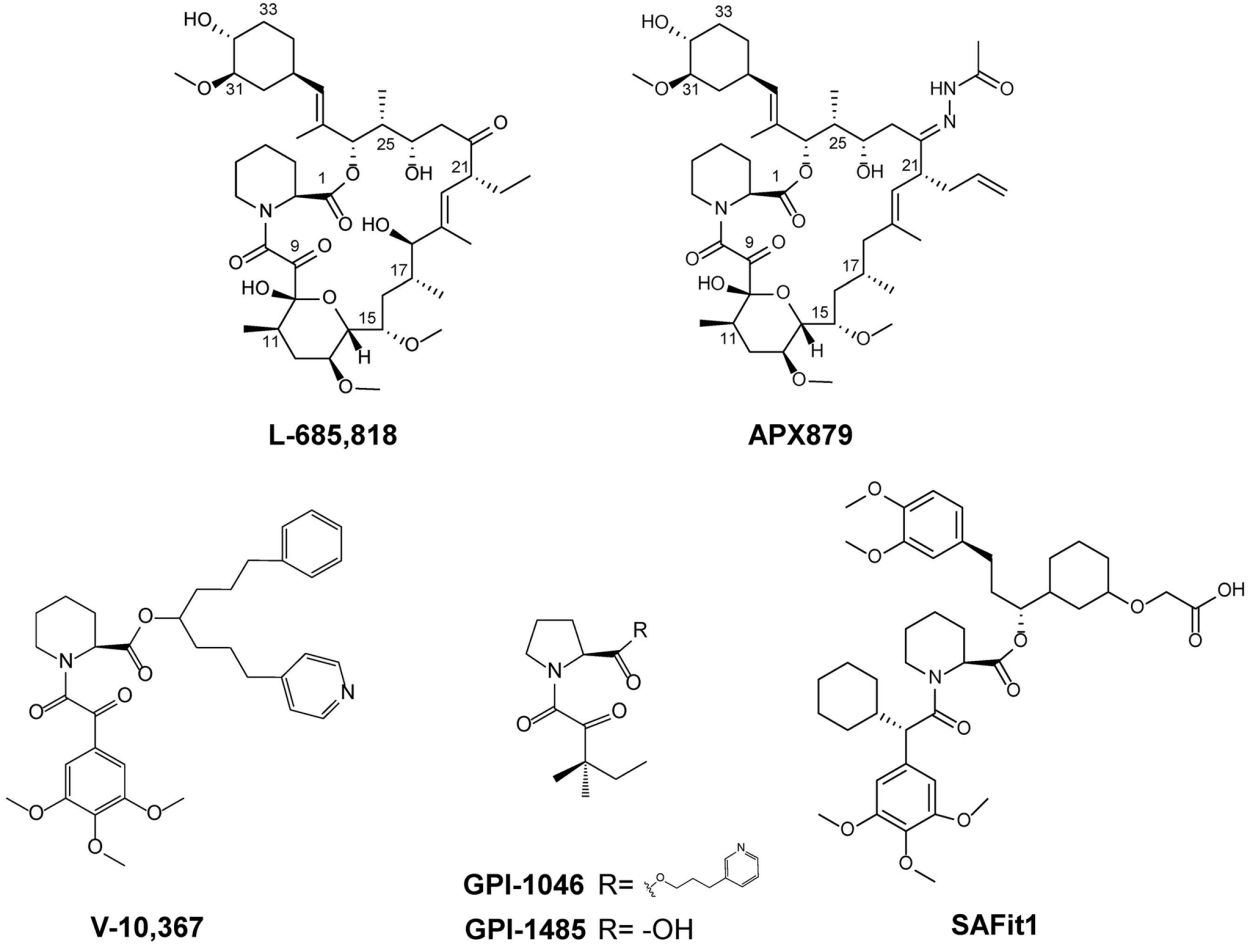
Non-immunosuppressive FKBP ligands generated by chemical synthesis. L-685,818, and APX879 exhibited antifungal activity. L-685,818, V-10,367, GPI-1046, GPI-1485, and SAFit1 showed neuronal activities.

The structure of the human FKBP12–FK506–CaN ternary complex related to immunosuppressive activity was revealed decades ago [32], but the fungal FKBP12–FK506– CaN ternary complex has recently been identified [33]. Nevertheless, substantial efforts have been made to develop FK506 analogs that possess reduced immuno-suppression but retain potent antifungal activity before the fungal FKBP12–FK506–CaN ternary complexes were identified. L-685,818, which was generated by chemical synthesis, is a less immunosuppressive FK506 analog with antifungal activity (Fig. 3). This analog has hydroxyl group at C18 and ethyl side chain at C21. Due to these differences, L-685,818 showed slightly lower antifungal activity than FK506 against *C. neoformans* but its immunosuppressive activity was significantly reduced (10^5^-fold reduction) [11]. In addition, it was toxic against *C. neoformans* at 37°C but not at 24°C, like FK506 [34]. However, L-685,818 did not show antifungal activity against *A. fumigatus*, either alone or with caspofungin [31, 35].

APX879 appended with acetohydrazide at C22 of FK506 is also a chemically modified analog [33] (Fig. 3). This compound also exhibited approximately 71-fold reduced immunosuppression activity compared to FK506 but showed potent antifungal activity against *C. neoformans*, *C. albicans*, and *A. fumigatus*. This is because the acetohydrazide chain at C22 of APX879 interacts less favorably with His88 residue of mammalian FKBP12 compared to the corresponding Phe88 of *A. fumigatus* FKBP12. The in vitro antifungal activity of APX879 against the three fungal pathogens was inferior to that of FK506, but it was less toxic and showed better efficacy than FK506 in the cryptococcal murine model of invasive fungal infection. Furthermore, combination treatment of APX879+ fluconazole was more effective than single treatment of APX879 or fluconazole in the cryptococcal infection murine model. However, the structural complexity of FK506 makes it difficult to chemically synthesize FK506 analogs. Therefore, combinatorial biosynthetic approaches involving the manipulation of FK506 biosynthetic genes could be a viable alternative to generate various biologically improved or modified FK506 analogs.

**Fig. 4:**
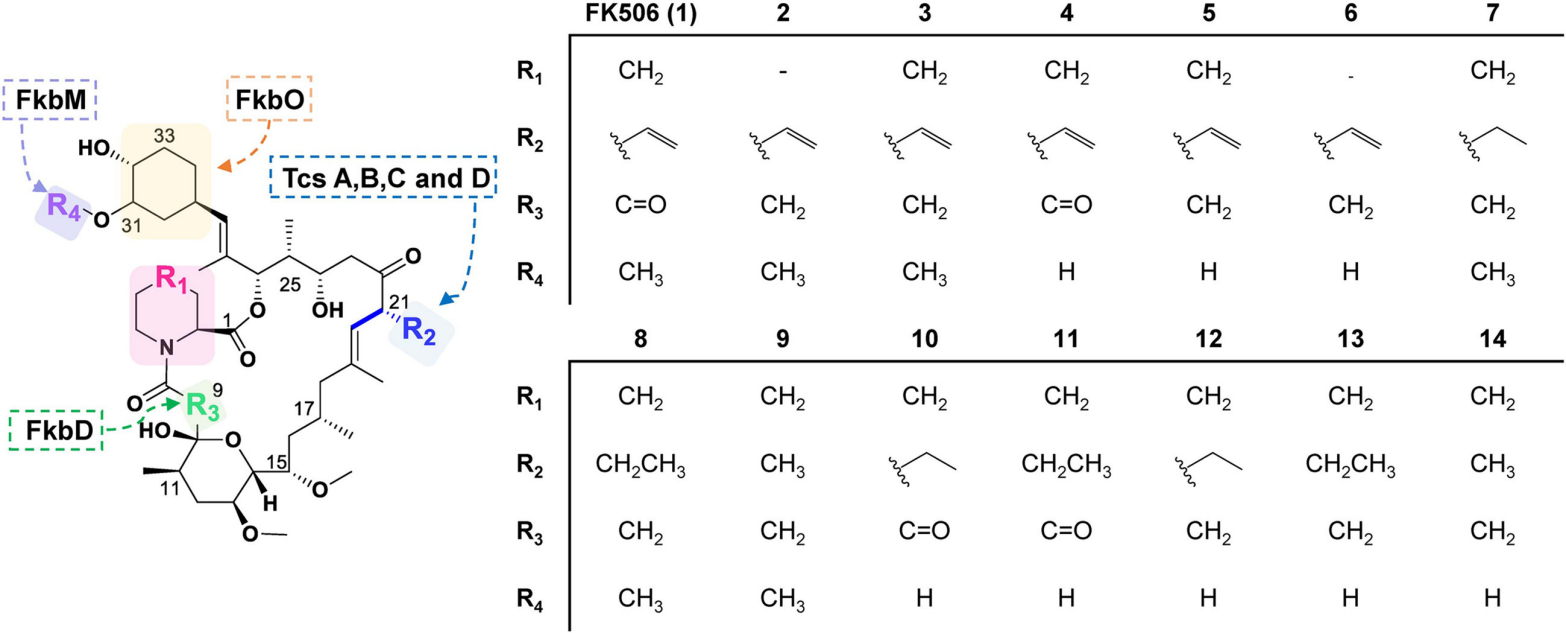
Representative structures of FK506 derivatives produced by combinatorial biosynthesis. FK506 derivatives were generated in eight deletion mutants: Δ*fkbD* (**2** and **3**), Δ*fkbM* (**4**), Δ*fkbD*M (**5** and **6**), Δ csD-Δ*fkbD* (**7**), Δ csB-Δ*fkbD* (**8** and **9**), Δ csD-Δ*fkbM* (**10** and **11**), Δ csD-Δ*fkbD*M (**12**), and Δ csB-Δ*fkbD*M (**13** and **14**).

For recent examples, four FK506 analogs, 9-deoxo-prolylFK506 (**2**), 9-deoxoFK506 (**3**), 31-*O*-demethylFK506 (**4**), and 9-deoxo-31-*O*-demethylFK506 (**5**), were generated by modifying post-PKS tailoring genes involved in C9 oxidation and C31 methylation in *Streptomyces* sp. KCTC 11604BP [16, 36, 37] (Fig. 4). These compounds were first isolated to elucidate the biosynthetic pathway of FK506; however, they were found to have significantly reduced immunosuppression activity (9.1 to 9937-folds) while retaining potent antifungal activity. Despite lower antifungal activity than that of FK506, all these compounds, except for 9-deoxo-prolylFK506, showed a certain degree of antifungal activity against the aforementioned three major fungal pathogens. In addition, these analogs exhibited synergistic antifungal activity with other commercial antifungal drug fluconazole. Moreover, it was proven that FK506 analogs, like FK506, exerted antifungal effects by inhibiting the CaN pathway because they did not show antifungal activity when the CaN subunit was inactivated in *C. neoformans* and *C. albicans*. Among the four aforementioned FK506 derivatives, 9-deoxo-31-*O*-demethylFK506 (**5**) was selected as a new antifungal candidate on considering both immunosuppressive and antifungal activities, and its in vivo antifungal efficacy was evaluated. Remarkably, combination treatment of 9-deoxo-31-*O*-demethylFK506 (**5**)+ fluconazole significantly extended the survival of the infected mice, suggesting that C9 and C31 positions in the FKBP-binding region could be potential sites to control the binding affinity of FK506 to human and fungal FKBP12 [37]. 

To lower the immunosuppressive activity further while maintaining the antifungal activity, nine FK506 analogs containing modified C1, C9, C21, and C31 were recently biosynthesized by combinatorially deleting the allylmalonyl-CoA synthetic gene *tcsB* or *tcsD*, which are involved in the formation C21 side chain, in addition to deleting *fkbD* or *fkbM*, which are involved in the post-PKS modification steps at C9 and C31 positions [38]. Seven compounds, including 9-deoxo-31-*O*-demethyl-prolylFK506 (**6**), 9-deoxo-36,37-dihydroFK506 (**7**), 9-deoxoFK523 (**9**), 31-*O*-demethyl-36,37-dihydroFK506 (**10**), 9-deoxo-31-*O*-demethyl-36,37-dihydroFK506 (**12**), 9-deoxo-31-*O*-demethylFK520 (**13**), and 9-deoxo-31-*O*-demethylFK523 (**14**) were new, and 9-deoxoFK520 (**8**) [39, 40] and 31-*O*-demethylFK520 (**11**) [41, 42] were already known (Fig. 4). The structure–activity relationship (SAR) was investigated by assessing immuno-suppression and antifungal activity. Compared to 9-deoxo-31-*O*-demethylFK506 (**5**), C9 and C21 modified analogs generally showed similar or much lower immunosuppressive activity and were more advantageous to maintain strong antifungal activity, except 9-deoxoFK523 (**9**), which has the methyl side chain at C21. 31-*O*-demethyl-36,37-dihydroFK506 (**10**) and 31-*O*-demethylFK520 (**11**), which are modified at both positions of C31 and C21, exhibited high antifungal activity but also showed high immunosuppressive activity. Moreover, C1, C9, C31, and C21 modified analogs showed significantly reduced immunosuppression but no noticeable antifungal activity. Importantly, a combination of fluconazole with FK506 analogs modified with C9 and C21 further enhanced antifungal activity. Consequently, 9-deoxoFK520 (**8**) has the potential to be a new antifungal agent against a broad range of pathogens [38].

## Putative Mechanism of FK506 for Neuroprotection and Neuroregeneration

FK506 has various neurological activities, such as neuro-protection in focal cerebral ischemia model of stroke [9] and neuroregeneration in animal models of neurodegenerative diseases, including nerve injury [43]. FK506 also enhances neurite outgrowth [10]. The neuronal mechanism of FK506 is unclear, but neuronal properties of FK506 are unequivocally related to FKBP and perhaps even to CaN in part [44].

**Fig. 5:**
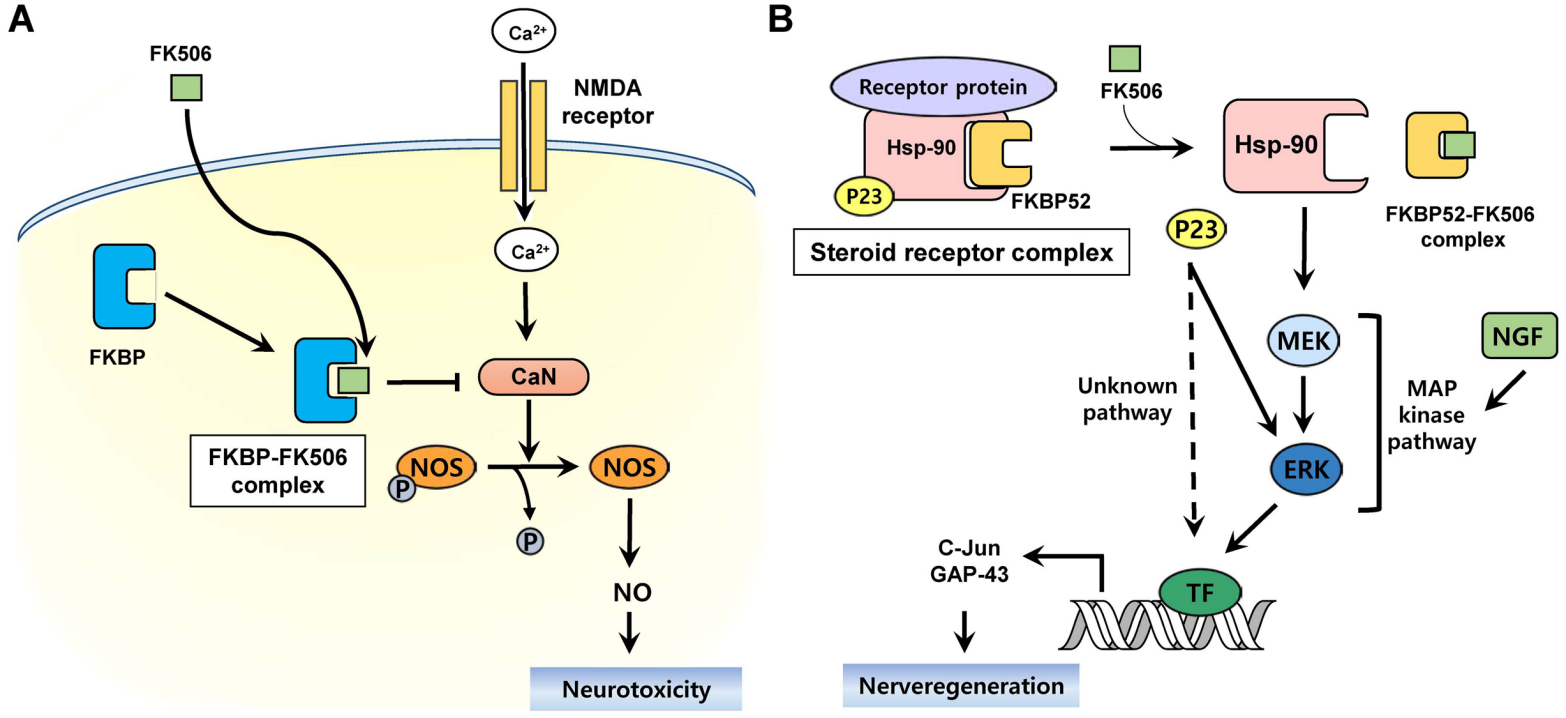
Proposed neurotrophic mechanism of action of FK506. FK506 is involved in neuroprotective (**A**) and neuroregenerative action (**B**).

The proposed neuroprotective mechanism of FK506 is shown in Fig. 5A [45]. Nerve damage, including cerebral infarction and degenerative disease, is associated with glutamate neurotoxicity, which exhibits toxicity through *N*-methyl-D-aspartate (NMDA) receptors [46]. However, preventing nitric oxide (NO) production associated with NMDA toxicity may have a protective effect against this neurotoxicity [47]. NO synthase (NOS) can be phosphorylated by inhibiting CaN, thus preventing the formation of NO [48, 49]. Therefore, the FK506–FKBP complex exhibits neuroprotective activity by inhibiting CaN, dephosphorylation of NOS, and reduction of NO formation against glutamate toxicity in vitro [49]. Nonetheless, it is believed that an alternative mechanism may be involved in its neuroprotective effects because it was observed that NO inhibitors were not neuroprotective in vivo [50].

Regarding nerve regeneration activity, it has been reported that FKBP52 rather than FKBP12 is involved in the neuroregenerative action of FK506 [51, 52]. In neuronal hippocampal cultures, FK506 treatment showed comparable neuronal growth in FKBP12 knockout and wild-type mice (30% and 26%, respectively). In addition, it was confirmed that the FKBP52 antibody, which does not bind FKBP12, interferes with neurite outgrowth of FK506 [51]. Putative neuroregenerative action begins with the dissociation of steroid receptor complex comprising the steroid receptor protein, FKBP52, Hsp90, and p23 [53, 54] (Fig. 5B). FK506 binds to FKBP52 to dissociate the complex, which activates Hsp90 to release p23. Hsp90 stimulates nerve regeneration by activating the mitogen-associated protein (MAP) kinase pathway and then increases c-Jun and GAP-43 involved in nerve regeneration by the ERK pathway [55, 56]. Nerve growth factor (NGF) also synergistically activates the MAP kinase pathway along with FK506 to promote nerve regeneration [10]. P23 promotes nerve regeneration by the ERK pathway or unknown pathways. In summary, the FKBP52–FK506 complex mediates nerve regeneration by the activation of Hsp90 and dissociation of p23 [51-53].

In addition, FKBP51, which is structurally very similar to FKBP52, is recently known to be involved in neuro-regenerative activity [57-59]. It has been discovered that selective inhibition of FKBP51 can enhance neuroregeneration [59]. Notably, these two proteins have opposite effects on steroid hormone receptors [60, 61]. Therefore, it is important to develop FK506 derivatives that can distinguish between FKBP51 and FKBP52. Furthermore, FKBP52 may cause reproductive problems, but FKBP51 may be effective in treating depression [62-64]. FKBP51 and FKBP52 have similar affinity to FK506 [65, 66], high homology [67, 68], and similar active sites [69]; thus, it is difficult to identify the true target of the non-immunosuppressive FKBP ligands developed before 2014 [70, 71].

## FK506 Analogs with Neuronal Properties

It has been reported that immunosuppressive activity of FK506 can be separated from its neuronal activity [72]. The chemically synthesized V-10,367, which lacks the right side of FK506, is a representative FKBP12 inhibitor with significantly reduced immunosuppressive activity [73] (Fig. 3). It has shown neuroprotective activity in NTP-methyl-4-phenyl-1,2,3,6-tetrahydropyridine (MPTP)-treated mouse and rat models of Parkinson’s disease [74] and has shown functional recovery, neuronal regeneration, and neurite growth activity [72]. L-685,818, mentioned in the previous antifungal section, has also showed neuronal regeneration activity [75]. GPI-1046, which has >5000-fold-reduced immunosuppression activity compared to FK506, has exhibited neuronal properties, including neuroprotection, neurite outgrowth, and nerve recovery, and has become a clinical candidate for the treatment of Parkinson's disease [76, 77] (Fig. 3). Since then, several studies have suggested FKBP52 as the target of neuronal regeneration [51, 52, 77], and GPI-1485, one of the GPI-1046 series, was studied up to phase 2 as a non-FKBP12 inhibitor but failed due to lack of clinical benefit [57, 78, 79] (Fig. 3). Nevertheless, the development of FKBP inhibitors is ongoing, and as mentioned above, it has become particularly important to distinguish between FKBP51 and FKBP52 as neuronal regeneration targets. The recently synthesized SAFit1 targets only FKBP51, did not affect the production of IL-2, which causes an immune response, and promoted neurite growth [70] (Fig. 3).

**Fig. 6:**
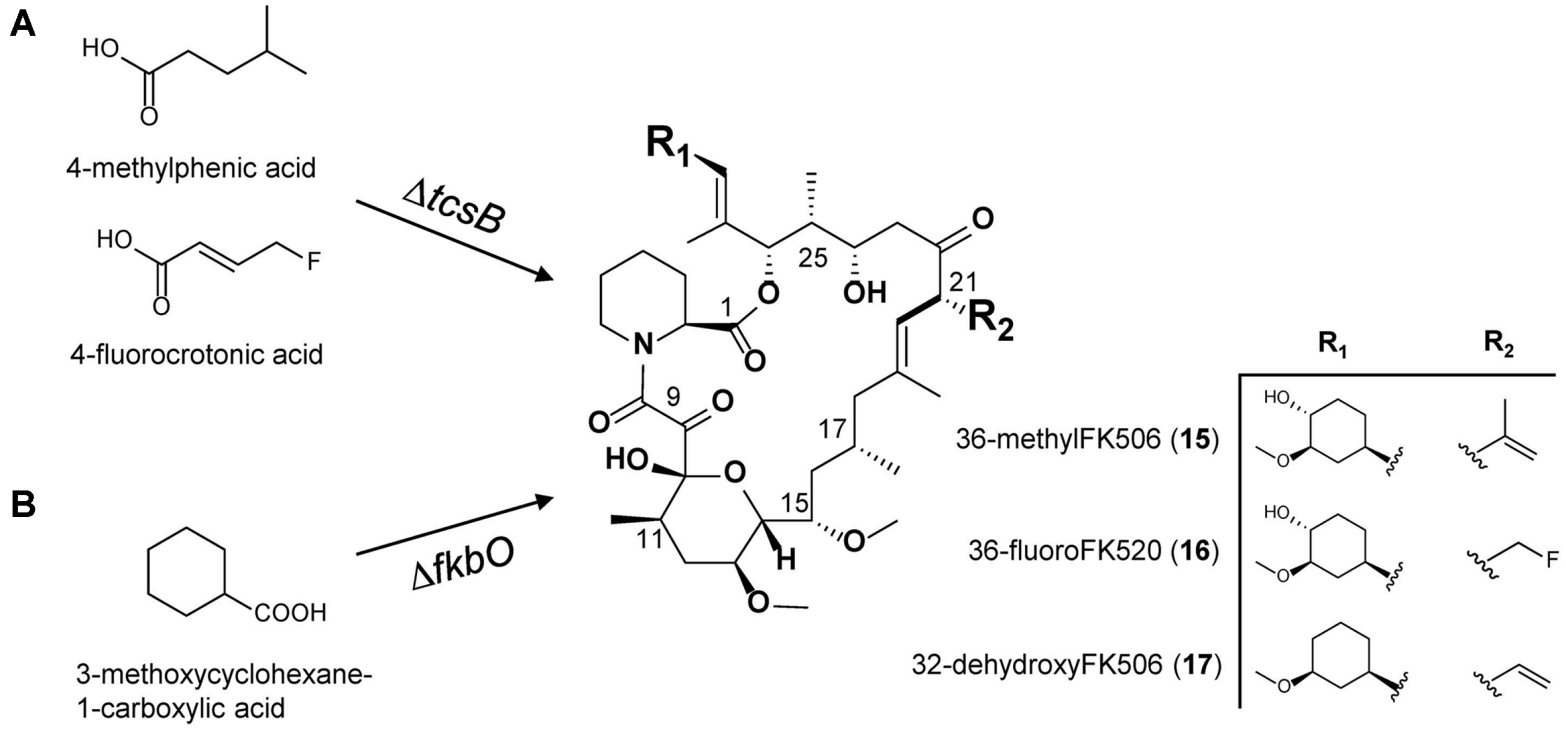
Mutasynthesis of FK506 derivatives in Δ*tcsB* or Δ*fbO* strain with chemical feeding. (**A**) 36-methyl-FK506 (**15**) and 36-fluoro-FK520 (**16**) were obtained from the culture of Δ csB strain supplemented with 4-methylphenic acid and 4-fluorocrotonic acid, respectively. (**B**) 32-dehydroxy-FK506 (**17**) was obtained from the culture of Δ*fkbO* strain supplemented with 3-methoxycyclohexane-1-carboxylic acid.

FK506 derivatives with reduced immunosuppressive and neuronal regenerative activities have also been developed by the modification of the FK506 biosynthetic pathway. For example, new FK506 derivatives were generated by mutational biosynthesis. 36-MethylFK506 (**15**) and 36-fluoroFK520 (**16**) were produced when 4-methylpentanoic acid and 4-fluorocrotonic acid were fed to the *tcsB* mutant, respectively (Fig. 6A). Both these substances showed neurite outgrowth, albeit with slightly reduced immunosuppressive activity. In particular, neurite outgrowth of 36-methylFK506 (**15**) increased 1.2-fold compared to FK506 [14]. 32-DehydroxyFK506 (**17**) is also an FK506 derivative produced by feeding 3-methoxycyclohexane-1-carboxylic acid to the deletion mutant of *fkbO*, which is involved in the biosynthesis of the starter unit of FK506. This compound reportedly shows reduced immunosuppression by three-fold and slightly increased neurite outgrowth activity than FK506 [80] (Fig. 6B). 9-Deoxo-prolylFK506 (**2**), which was obtained through the deletion of *fkbD*, exhibited slightly reduced neuronal regeneration activity but significantly reduced immunosuppression [36] (Fig. 4).

## Summary and Future Perspectives

The strong immunosuppressive activity of FK506 should be reduced or eliminated to harness the other useful effects, such as antifungal and neurotrophic activities. Several studies have shown that these activities can be separated from immunosuppressive activity and maintained. FKBP12 and CaN were found to be important targets for immunosuppressive and antifungal activity of FK506 [8], and chemically synthesized FK506 derivatives showed the possibility of separation of immunosuppressive and antifungal activities [11, 33]. In addition, various FK506 derivatives were developed through combinatorial biosynthesis and showed the possibility of modulating the immunosuppressive and antifungal activity by modifying some specific functional groups of FK506 [37, 38]. The structure of fungal FKBP12–FK506–CaN, which has been recently discovered, will provide a valuable insight to structure-guided fungal-specific inhibitor design [33].

The mechanisms of neuroprotective and neuronal regeneration activity of FK506 are unclear. In the early studies, chemically synthesized FK506 derivatives showed that neuronal activity could be separated from immuno-suppressive activity [73, 76] and that neuronal regeneration was related to FKBP52 as they exhibited neuronal regeneration property without binding to FKBP12 [51, 52]. FKBP51 was recently suggested to be a true target for neuronal regeneration. Moreover, while FKBP52 may cause reproductive function [62, 63], FKBP51 is a safe target for neuronal regeneration and stress-related disorders, such as depression [58, 64]. Although these two proteins were difficult to distinguish due to their common features, SAFit derivatives showed the possibility of distinguishing these two proteins [70]. FK506 derivatives made to date through biosynthesis showed significantly reduced immuno-suppression while maintaining comparable neuroregenerative activities as FK506. However, it is not certain if these derivatives can distinguish between FKBP51 and FKBP52 [14, 36, 80]. Therefore, considering the advantages of combinatorial biosynthesis, which enables biosynthesis of diverse structures, detailed SAR and target identification studies of various derivatives in the future are expected to increase the possibility to develop a new therapeutic agent for neurological diseases.
